# Nanostructured (Co, Mn)_3_O_4_ for High Capacitive Supercapacitor Applications

**DOI:** 10.1186/s11671-017-1977-0

**Published:** 2017-03-23

**Authors:** Qinghua Tian, Xiang Wang, Guoyong Huang, Xueyi Guo

**Affiliations:** 1 School of Metallurgy and Environment, Central South University, 410083 Changsha, China; 2 Cleaner Metallurgical Engineering Research Center, Nonferrous Metal Industry of China, 410083 Changsha, China

**Keywords:** Manganese oxide, Cobalt doping, Supercapacitor, Ozone, Nanogranules

## Abstract

Nanostructured Co doped Mn_3_O_4_ spinel structure ((Co, Mn)_3_O_4_) were prepared by co-precipitation under O_3_ oxidizing conditions and post-heat treatment. The product was composed of nanogranules with a diameter of 20–60 nm. The electrochemical performance of (Co, Mn)_3_O_4_ electrode was tested by cyclic voltammetry, impedance, and galvanostatic charge-discharge measurements. A maximum specific capacitance value of 2701.0 F g^−1^ at a current density of 5 A g^−1^ could be obtained within the potential range from 0.01 to 0.55 V versus Hg/HgO electrode in 6 mol L^−1^ KOH electrolyte. When at high current density of 30 A g^−1^, the capacitance is 1537.2 F g^−1^ or 56.9% of the specific capacitance at 5 A g^−1^, indicating its good rate capability. After 500 cycles at 20 A g^−1^, the specific capacitance remains 1324 F g^−1^ with a capacitance retention of 76.4%.

## Background

Supercapacitors have several advantages, such as long cycle life, high power density, high specific capacitance (1000 F g^−1^), and environmental friendliness, which are widely used in various fields such as electric vehicles, starting power of fuel cells, and new energy equipments. [1–4]. The electrode materials used in double-layer capacitors (EDLCs) usually are carbon-based materials, but they often suffer from their low capacitance. To improve the specific capacitance of supercapacitors, lots of researches have been dedicated to the investigation of transition metal oxides materials, owing to their several oxidation states [5–10]. Hydrous ruthenium oxide was founded to be an excellent electrode material due to its remarkable high specific capacitance and excellent reversibility, but the extremely high cost restricted its commercial application. Therefore, considerable efforts have been devoted to studying on inexpensive transition metal oxides like MnO_2_ and Co_3_O_4_ [11–15]. Among these metal oxides, Co_3_O_4_ is reported to be one of the most potential materials due to its high redox activity, good reversibility, and high theoretical specific capacitance (3560 F g^−1^) [16–18]. Manganese oxide is also a very promising material, due to its excellent cycling stability, low cost, and environmental friendliness [19, 20].

To make full use of the advantage of each potential material, multinary metal oxides were designed to be synthesized. And it has been proven that preparing compounds of mixed oxides composites is an effective way to obtain superior capacitive performance as the electrode [21, 22]. Therefore, Co-Mn oxides have attracted the interest of researchers and attention of the industry [23–25]. For example, Kong et al. [18] prepared Co-Mn composite oxide (spinel MnCo_2_O_4_) powder by a sol–gel method, showing that the specific capacitance was 405 F g^−1^ at 5 mA cm^−2^, and the capacitance retention ratio was 95.1% after 1000 cycles. Zhao et al. [26] synthesized Mn-Co oxide nanowire with a specific capacitance of 396 F g^−1^ and found that manganese oxide-specific capacitance could be increased by doping cobalt ions. Chang et al. [27] prepared Mn-Co oxides with a specific capacitance of 186 F g^−1^ by anodic deposition and showed that Co addition could hinder the dissolution of Mn into electrolyte, which enhanced the reversibility and stability of cobalt-manganese oxide composite. However, the specific capacitances of these materials are still quite low, further study of the Mn-Co multinary oxide is needed. To the best of our knowledge acquired, there are no reports on preparation of Co doped Mn_3_O_4_ spinel structure by a gas-liquid reaction for supercapacitors. Besides, the preparation methods always are sol–gel, hydrothermal method, and electrodeposition, which need a long reaction time. It is very necessary to explore a new method which is simple and suitable for large-scale preparation.

Herein, in this study, we synthesized (Co, Mn)_3_O_4_ nanogranules composite using a method of co-precipitation under O_3_ oxidizing conditions followed by post-heat treatment. This precipitation process has very high production efficient within 1 h. The crystal structure and morphology are investigated. And the electrochemical tests show that the (Co, Mn)_3_O_4_ electrode demonstrated a large specific capacitance and a long cycle life.

## Methods

(Co, Mn)_3_O_4_ were prepared as follows: equimolar amount of Co and Mn acetate was dissolved in 200 mL deionized water (each salt concentration was set to 12.5 mmol/L^−1^, respectively), sulfuric acid was subsequently added in the solution to adjust pH = 3.5 under constant stirring. Ozonizer (OZOMJB-10B, ANQIU OZOMAX EQUIPMENT) provided a constant amount of ozone during the whole experiment, and the gas (a mixture of ozone and oxygen) flow rate was controlled by a gas flow meter [28]. The gas was injected into the solution at 35 °C for 1 h at 2.0 L/min. The precipitated precursors were separated by centrifugation and were washed several times with deionized water and ethanol. The precursors were dried in a vacuum oven at 80 °C for 4 h, and then the products were calcined in air at 600 °C for 2 h (post-heat treatment) to obtain the (Co, Mn)_3_O_4_ nanogranules.

The crystal phases of the composites were characterized by X-ray diffractometer (XRD, Rigaku TTR III). The chemical states of Mn and Co in the samples were carried out using X-ray photoelectron spectroscopy with Al Ka radiation (XPS, Thermo ESCALAB250Xi). Scanning electron microscope (SEM, Nova NanoSEM230) and transmission electron microscopy (TEM, JEM-2100F) were employed to characterize the morphology and nanostructures.

To prepare the working electrodes, 75 wt.% active material((Co, Mn) _3_O_4_ nanogranules), 20 wt.% acetylene black, and 5 wt.% polytetrafluoroethylene (PTFE) binder were mixed into water, and then the mixture was coated onto 1.5 × 1 cm^2^ Ni foam within an area of 1 × 1 cm^2^, which were then dried in vacuum at 80 °C for 4 h to remove the solvent. The mass loading of (Co, Mn)_3_O_4_ nanogranules was about 1.93 mg cm^−2^. A Pt-sheet was employed as the counter electrode, and a Hg/HgO electrode was used as the reference electrode. All electrochemical tests were carried out in the three-electrode system in 6 mol L^−1^ KOH solution by an Autolab PGSTAT302N electrochemical workstation.

## Results and Discussion

**Fig. 1:**
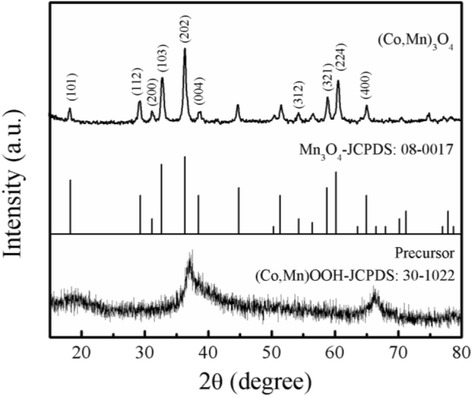
XRD patterns of the precursor and (Co, Mn)_3_O_4_ nanospheres

The XRD pattern of the precursor and the final product after annealing at 600 °C for 2 h were depicted in Fig. 1, respectively. The diffraction peaks of the precursor are indexed to (Co, Mn)OOH (JCPDS no.30-1022), and the reflection peaks of final product are well matched with hausmannite Mn_3_O_4_ (JCPDS no.08-0017). It can be seen that the position of diffraction peaks of the sample is slightly right shifted compared to the standard hausmannite Mn_3_O_4_. The diffraction peaks at 18.2°, 29.3°, 31.1°, 32.7°, 36.3°, 38.4°, 54.3°, 58.7°, 60.2°, and 65.0° can be indexed as (101), (112), (200), (103), (202), (004), (312), (321), (224, and (400) crystal plane. No characteristic peaks peculiar to impurities of other crystalline phases, such as Co_3_O_4_ and MnO_2_ are observed. It suggests that doped cobalt ions have been well incorporated into the Mn lattice site without distorting the crystal symmetry. The lattice parameters a, b, and c of the product are 5.73 Å, 5.73 Å, and 9.52 Å, respectively, which are close to the values of Mn_3_O_4_ (a = 5.76 Å, b = 5.76 Å, and c = 9.44 Å according to JCPDS no.08-0017). The results can be attributed to the different ionic size of cobalt and manganese (ionic radii of Co^2+^−0.65 Å, Mn^2+^−0.67 Å, Co^3+^−0.61 Å, Mn^3+^−0.65 Å). After being calcined at 600 °C, all diffraction peaks present a sharp state, which reveals good crystallization of the (Co, Mn)_3_O_4_ powder.

**Fig. 2:**
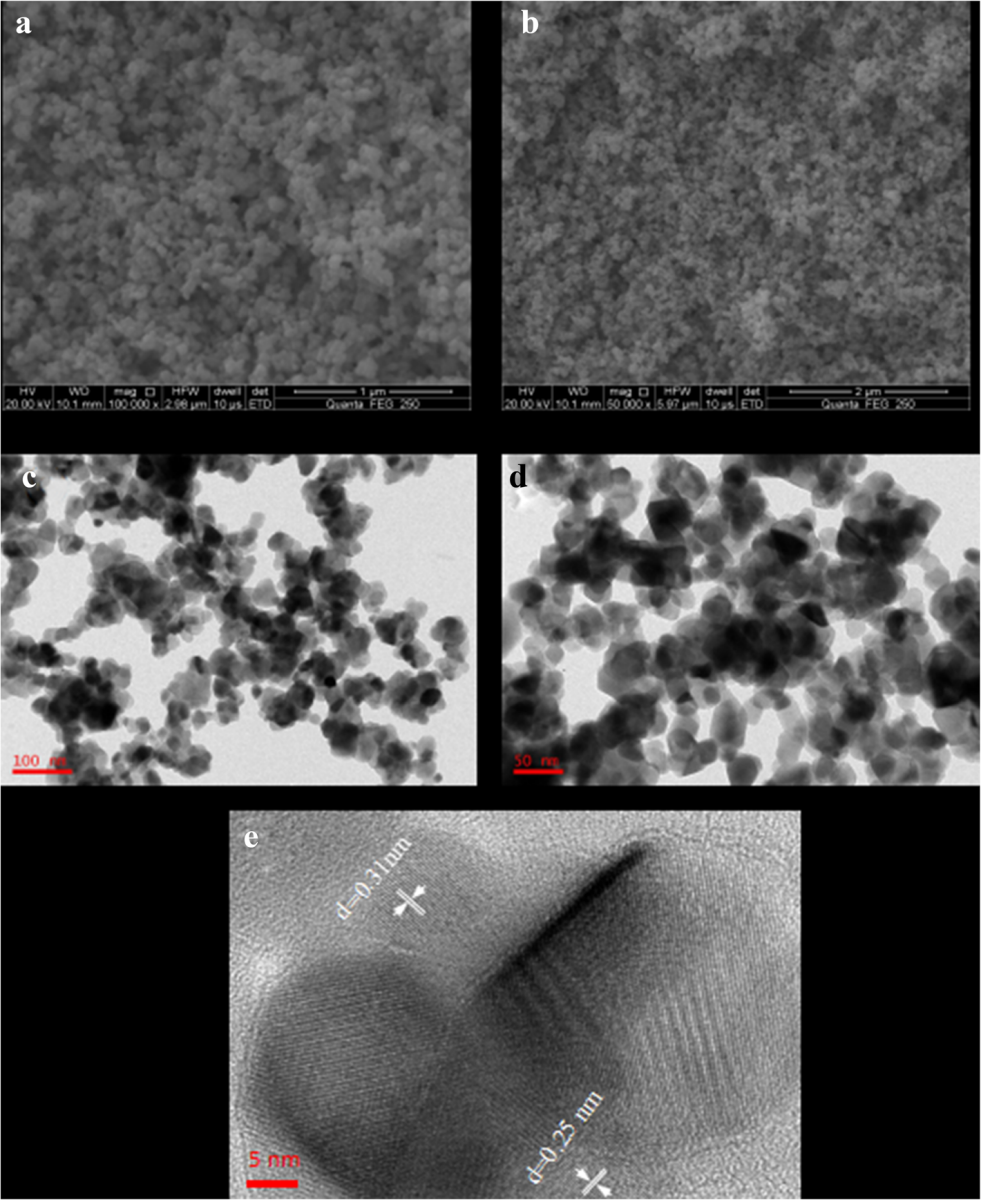
**a** *High*-magnification and **b** *low*-magnification SEM, **c** *low*-magnification and **d** *high*-magnification TEM, **e** HRTEM images of (Co, Mn)_3_O_4_

The SEM images of (Co, Mn)_3_O_4_ with different magnification presented in Fig. 2a, b demonstrate that the powders show a loosely packed porous structure and ball-like morphology, whose particle size is small and looks well-dispersed. Numerous macropores and mesopores exist, and it is well-documented that macropores can serve as ion buffering reservoirs and mesopores are capable of overcoming the primary kinetic limits of electrochemical processes [18, 29]. Through TEM with different magnification in Fig. 2c, d, it can be clearly seen that these particles are nanoscale granules with a diameter of 20–60 nm, providing high surface area which is beneficial to high capacitive supercapacitor. Furthermore, the measured lattice spacing from HRTEM image are 0.31 and 0.25 nm, corresponding to the interplanar distance of the (112) and (202) planes of hausmannite Mn_3_O_4_ phase, respectively. This implies the well-dispersion of Co into the lattice of Mn_3_O_4_, and that also excluded conglomeration of CoOx, The substitution of Co has little effect on the crystal structure of hausmannite Mn_3_O_4_.

**Fig. 3:**
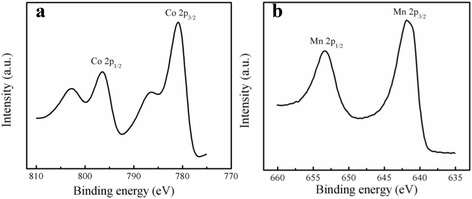
XPS data in Co (**a**) and Mn (**b**) regions for (Co, Mn)_3_O_4_ electrode

XPS was conducted to analyze the chemical valence of Co and Mn in the (Co, Mn)_3_O_4_ nanogranules. XPS surveys of the composite oxide were presented in Fig. 3. According to quantitative XPS analysis, atomic concentrations of cobalt and manganese were approximated 1:4.5 (Co:Mn = 5.87:26.39). There were two main peaks at 780.3 and 795.8 eV corresponding to Co 2p3/2 and Co 2p1/2, and two additional satellite peaks located at 786.5 and 802.8 eV, which are the characteristics of Co^2+^ and Co^3+^ ions, respectively [15]. The first satellite peak was 6.2 eV above the Co 2p3/2 peak, and second satellite peak 7 eV was above the Co 2p1/2 main peak. It is reasonable to determine that Co^2+^ and Co^3+^ exist on the surface of the materials according to the binding energy and energy separations of satellites peaks [30–32]. The Mn 2p spectra exhibits two major peaks at 641.9 and 653.5 eV, which supports the presence of Mn^2+^ and Mn^3+^ species according to the literatures reporting the binding energy values associated to manganese oxides [33]. The XRD and XPS results support the formation of the structured (Co, Mn)_3_O_4_ by this method. More importantly, cobalt and manganese cations with relatively similar oxidation states share the lattice sites.

**Fig. 4:**
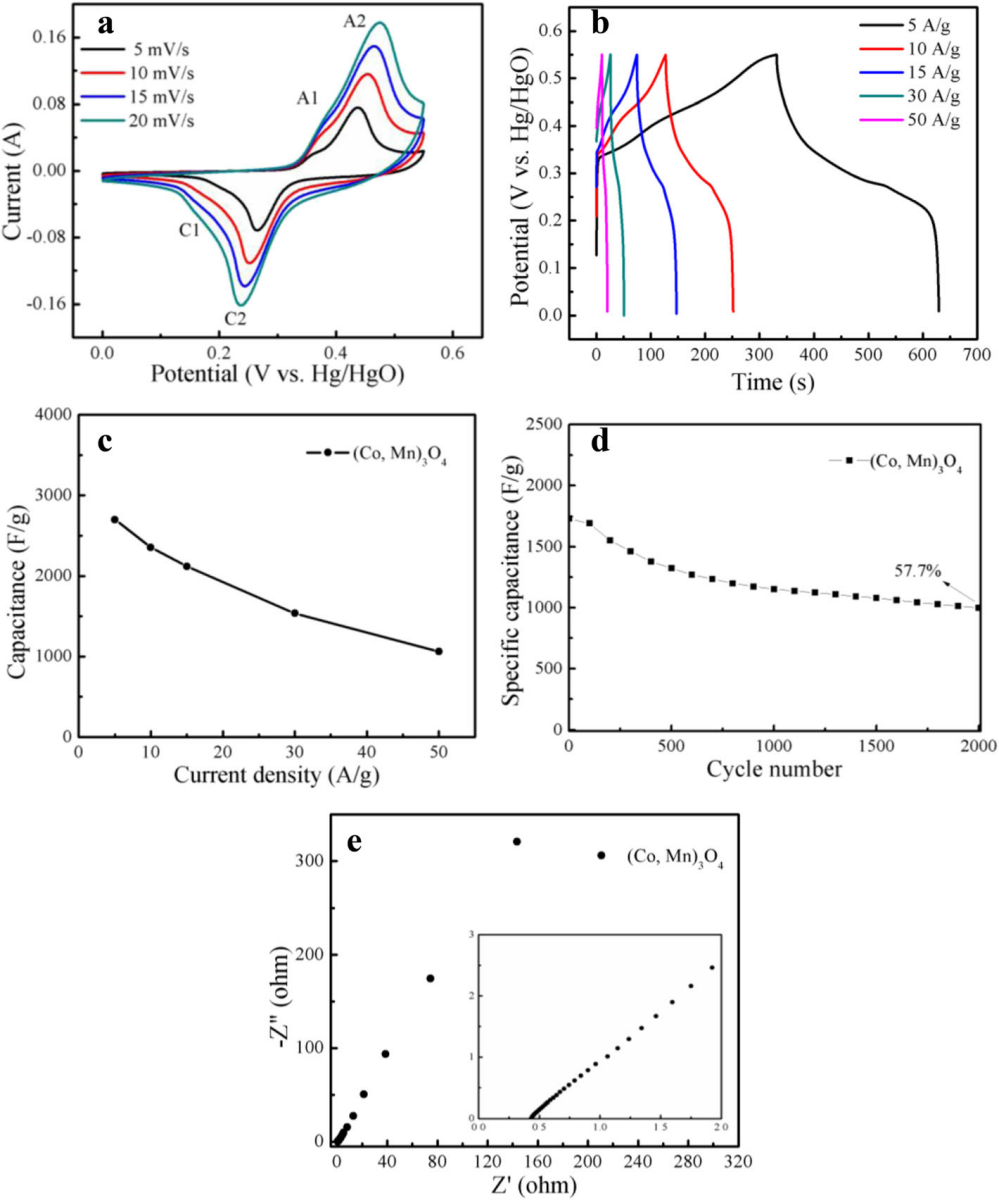
**a** CV *curves* of the (Co, Mn)_3_O_4_ electrode at different scan rates in the potential range of 0–0.55 V. **b** The discharge *curves* of the (Co, Mn)_3_O_4_ electrode at different current densities. **c** Dependences of the discharge specific capacitance on the charge/discharge current densities. **d** Specific capacitance of (Co, Mn)_3_O_4_ electrode materials as a function of cycle number recorded at a current density of 20 A g^−1^. **e** Nyquist *plots* of different (Co, Mn)_3_O_4_ electrodes in a frequency range from 10^-2^ ﻿ to 10^5^ Hz measured (*inset*: the magnification of the high frequency region of the impedance spectra)

Cyclic voltammetry tests have been conducted at different scan rates in a fixed potential range of 0–0.55 V (vs. Hg/HgO) (Fig. 4a). Obvious anodic peaks (A1 and A2) and cathodic peaks (C1 and C2) can be observed, indicating that the energy storage mainly comes from the Faradic redox reaction of both Mn and Co. The eletrochemical behavior of Co-Mn composites between different oxidation states [34, 35] results from the following redox reactions [19]:

1 $$ {\left(\mathrm{Co},\mathrm{Mn}\right)}_3{\mathrm{O}}_4+{\mathrm{H}}_2\mathrm{O} + \mathrm{O}{\mathrm{H}}^{-} - {\mathrm{e}}^{-} = \mathrm{CoOOH} + \mathrm{MnOOH} $$

2 $$ {\left(\mathrm{Co},\mathrm{Mn}\right)}_3{\mathrm{O}}_4+{\mathrm{H}}_2\mathrm{O} + {\mathrm{O}\mathrm{H}}^{-} - {\mathrm{e}}^{-} = \mathrm{CoOOH} + \mathrm{MnOOH} $$

A1/ C1 couple suggests the redox of Mn^2+^/Mn^3+^ and Co^2+^/Co^3+^, A2 /C2 couple indicates the redox of Mn^3+^/Mn^4+^ and Co^3+^/Co^4+^. The peaks potential difference (*Δ*E = *E*_a_ − *E*_c_) at 5 mV s^−1^ of A1/C1 and A2/C2 were 150 and 170 mV, respectively, which are much smaller than that of the pure Co_3_O_4_ (290 and 270 mV at 5 mV s^−1^) [19], demonstrating that (Co, Mn)_3_O_4_ electrode exhibits more excellent electrochemical reversibility. All the CVs are approximate symmetry in anodic and cathodic directions and showing large current response, revealing good reversible and high capacitive behavior of the electrode.

The shape of CV curves deviated from the ideal rectangular shape of electric double layer reveal the obvious feature of faradic capacitance, displaying strong redox behavior. The redox peaks shifted with the increase of the scan rate, indicating the quasi-reversible feature of the redox couples, which is related to intercalate mechanism of the OH^−^ ions at the interface of electrode/electrolyte under higher scan rate [11]. Furthermore, the peak current densities increased with the increase of the scan rates from 5 to 20 mV s^−1^, which suggests its good reversibility of fast charge/discharge response.

The galvanostatic charge/discharge curves of the samples at various current densities (5 ~ 50 A/g^−1^) within a potential range of 0.01–0.55 V (vs. Hg/HgO) was shown in Fig. 4b. The charge curve was consisting of two segments. A linear variation with time (0.01–0.33 V) parallel to the vertical axis shows the typical characteristic of electric double layer capacitance and a sloped variation with time (0.33–0.55 V) is due to the redox process [36–38] of (Co, Mn)_3_O_4_. It can be identified from the charge/discharge profiles that the major type of charge storage behaviors in as synthesized electrode materials were based on Faraday reactions which were in good consistence with the CV studies. The specific capacitance was calculated according to the following equation [19]:

3 $$ {C}_{\mathrm{m}}=\frac{I_d\times \varDelta t}{\varDelta V} $$

Where *C*_m_ (F g^−1^) is the specific capacitance, *I*_d_ (A g^−1^) is the discharge current, Δ*t* (s) is the discharge time, Δ*V* (V) is the discharge potential range.

Based on the equation above, the specific capacitances are 2701.0, 2356.7, 2120.9, 1537.2, and 1060.0 F g^−1^ at the current densities of 5, 10, 15, 30, and 50 A g^−1^, respectively. Apparently, the capacitive performance is much superior than that of many researchers. Xu et al. [19] prepared self-supported (Co, Mn)_3_O_4_ nanowires composite on nickel foam, which exhibited a specific capacitance of 611 F g^−1^ at a charge/discharge current density of 2.38 A g^−1^. Naveen et al. [15] synthesized manganese doped cobalt oxide nanoparticles exhibited a specific capacitance of 440 F g^−1^. The high specific capacitances may be attributed to its small size granules providing large reaction surface area, fast ion and electron transfer, and good electrochemical activity. Doping cobalt ions into the manganese oxide system may be an important factor in increasing the specific capacity. At a large current density of 30 A g^−1^, the specific capacitance of (Co, Mn)_3_O_4_ electrode maintains 1537.2 F g^−1^ or 56.9% of the specific capacitance at 5 A g^−1^, indicating its good rate capability. The characteristic of maintaining large capacitances under high charge/discharge current densities directly determines its excellent application prospects. The decrease of capacitance with the increase of current density as shown in Fig. 4c is likely caused by the increase of the ohmic drop due to electrode resistance and the relatively insufficient Faradic redox reaction of the active material under a higher discharge current density [39].

The electrochemical stability of the (Co, Mn)_3_O_4_ electrode was evaluated through the charge and discharge cycling test at the current density of 20 A g^−1^ for 2000 cycles. The trend of the specific capacitance versus cycle number is exhibited in Fig. 4d. It is found that the specific capacitance of the electrode materials gradually decreased during initial cycling tests then kept steady over a wide range of cycling numbers, showing an excellent reversibility of the charge/discharge processes and a very moderate fade rate at high current density. The specific capacitance of the (Co, Mn)_3_O_4_ after 500 and 2000 cycles are found to be 1324 and 998 F g^−1^ with capacitance retention of 76.4 and 57.7%, respectively. Compared to the reports by Kong et al. [18], Zhao et al. [26], and Chang et al. [27], the superior capacitance and good cycling indicate that the (Co, Mn)_3_O_4_ electrode has potential for application in supercapacitors. A tiny fraction of electroactive material falling off the nickel foam since the working electrode was immersed in electrolyte for a long time, and the irreversible Faraday reactions or the microstructure in the process of OH^−^ insertion (extraction) during oxidation (reduction) were possibly the main reasons for the specific capacitance loss.

Figure 4e shows the Nyquist plots of (Co, Mn)_3_O_4_ electrode at open circuit potential in 6.0 mol L^−1^ KOH solution. The frequency explored was from 10^−2^ to 10^5^ Hz, and Z’ and Z” are the real and imaginary parts of the impedance. Near absence of semicircle in the high frequency region depicts the low internal resistance of the electrode materials and diffusion controlled rate kinetics of the redox process [38], revealing fast electron transport through the (Co, Mn)_3_O_4_ electrode. The (Co, Mn)_3_O_4_ nanogranules have small dimensions (30–60 nm in diameter), providing short paths for electron transfer. The linear parts of the impedance spectra corresponding to Warburg impedance are typical for the capacitive response of the electrodes, which is dominated by the electrolyte diffusion process. The equivalent series resistance (ESR), which is composed of the ionic resistance of electrolyte, the intrinsic resistance of active materials, and the contact resistance between the active material and the current collector [40], is 0.43 Ω from the intercept at real axis at high frequencies. Naveen et al. [15] synthesized manganese doped cobalt oxide nanoparticles, which exhibited an ESR of 0.75 Ω. Kong et al. [18] prepared Co-Mn composite oxide, which exhibited an ESR of about 1 Ω. The lower ESR value indicates the higher electrical conductivity of the sample and higher utilization of energy during the charge/discharge process [41].

## Conclusions

In summary, we have synthesized successfully the (Co, Mn)_3_O_4_ composite nanogranules for the first time by oxidation precipitation with O_3_ and heat treatment. From TEM images, the diameter is about 20 ~ 60 nm, and the morphology of (Co, Mn)_3_O_4_ is advantageous to high performance electrochemical capacitor. Charge/discharge behaviors demonstated that (Co, Mn)_3_O_4_ nanogranules possessed high specific capacitances (2701.0 F g^−1^ at 5 A g^−1^ and 1060.0 F g^−1^ at 50 A g^−1^). And the materials also display a good cycling stability (after 500 cycles at 20 A g^−1^, the specific capacitance remains 1324 F g^−1^ with a capacitance retention of 76.4%). In consideration of the high preparation efficiency, high capacitance and simple preparation method, this (Co, Mn)_3_O_4_ nanogranule electrode has great potential applications in supercapacitor.

## Abbreviations

CV: Cyclic voltammetry
ESR: Equivalent series resistance
Fig: Figure
HRTEM: High resolution transmission electron microscopy
PTFE: Polytetrafluoroethylene
SEM: Scanning electron microscope
TEM: Transmission electron microscopy
XPS: X-ray photoelectron spectroscopy
XRD: X-ray diffraction
